# An ensemble framework for time delay synchronization

**DOI:** 10.1002/qj.3204

**Published:** 2018-01-15

**Authors:** Flavia R. Pinheiro, Peter Jan van Leeuwen, Ulrich Parlitz

**Affiliations:** ^1^ Department of Meteorology, University of Reading UK; ^2^ National Centre for Earth Observation (NCEO) Reading UK; ^3^ Max Planck Institute for Dynamics and Self‐Organization Göttingen Germany; ^4^ Institute for Nonlinear Dynamics, Georg‐August‐Universität Göttingen Germany

**Keywords:** synchronization, ensemble, time delay, data assimilation

## Abstract

Synchronization based state estimation tries to synchronize a model with the true evolution of a system via the observations. In practice, an extra term is added to the model equations which hampers growth of instabilities transversal to the synchronization manifold. Therefore, there is a very close connection between synchronization and data assimilation. Recently, synchronization with time‐delayed observations has been proposed, in which observations at future times are used to help synchronize a system that does not synchronize using only present observations, with remarkable successes. Unfortunately, these schemes are limited to small‐dimensional problems.

In this article, we lift that restriction by proposing an ensemble‐based synchronization scheme. Tests were performed using the Lorenz'96 model for 20‐, 100‐ and 1000‐dimension systems. Results show global synchronization errors stabilizing at values of at least an order of magnitude lower than the observation errors, suggesting that the scheme is a promising tool to steer model states to the truth. While this framework is not a complete data assimilation method, we develop this methodology as a potential choice for a proposal density in a more comprehensive data assimilation method, like a fully nonlinear particle filter.

## 1 Introduction

The synchronization phenomenon was first described back in the seventeenth century by Christiaan Huygens, a Dutch scientist who observed that two pendulum clocks suspended by a common frame were oscillating, after some time, in opposite directions but with the same frequency, independently of their start positions. He described further, that if an intervention was made to any of these oscillations, this agreement between the frequencies would be re‐established in a short period of time. The researcher attributed this ‘sympathy of two clocks’ to the frame they were sharing and its motion. Very recently, Peña‐Ramirez *et al.* (2016) presented a detailed study of Huygens' experiment and a mathematical model including the coupling of the clocks due to a wooden beam, confirming the occurrence of synchronization. Therefore, the main idea behind synchronization is that, if two (or more) systems share or exchange information in the correct way, they can synchronize to each other, i.e they can match some of their characteristics.

The equivalence between the concepts of synchronization and data assimilation was firstly pointed out by Duane *et al.* (2006). As stated by them, data assimilation aims to synchronize the model evolution with the true evolution of the system, finding the best estimate of the state and its evolution. The coupling is unidirectional, from the truth to the model system, and incomplete, as observations are typically sparse and contain errors. Typically, the model is extended with a nudging or relaxation term that forces it towards observations of the system. Synchronization then studies the stability of the synchronization manifold, which is the *D*‐dimensional linear subspace defined by **x**_S_=**x**_M_, where **x**_S_ and **x**_M_ denote states of the *D*‐dimensional system and the *D*‐dimensional model, respectively. To achieve synchronization, this manifold has to be transversally stable such that $\lim_{t\to \infty }\|x_{S}(t)-x_{M}(t)\|=0$. This stability is obtained with a suitable coupling term added to the model equations.

Recently, a coupling scheme exploiting delay embedding of the observations has been suggested, such that the forcing term also includes observations from either past or future (e.g. Abarbanel, 2013; Parlitz *et al.*, 2014; Rey *et al.*, 2014a, 2014b). These methods use the Jacobian of the forward model to move observation influence to the time instant of interest. A simplified approach has also been developed by Pazó *et al.* (2016), which avoids the calculation of the Jacobian, but at the expense of less strong synchronization. The main idea behind these methods is that observations from other times can be used to boost the number of observations in a system that would otherwise not synchronize. Remarkable synchronization results have been achieved this way by Rey *et al.* (2014a, 2014b), who performed tests considering a twin experiment using the Lorenz'96 chaotic model (Lorenz, 1995), with 20 state variables to be estimated and only one being observed. Among their results, they were able to find synchronization errors with magnitudes of the order of 10^−15^, close to machine precision. Furthermore, the authors achieved successful prediction ranges of around 2000 time steps. It should be mentioned, however, that their method assumes that the observations contain no errors, which is unrealistic in the data assimilation context. In their setting, even small observational errors can have a large impact on the synchronization results. Furthermore, their method is far too expensive to be used in realistic high‐dimensional geophysical problems as it needs the propagation of the Jacobian matrix of the forward model, of size the model dimension squared.

Nowadays, data assimilation is defined more generally as a Bayesian inference problem in which prior information about a system, described in a prior probability density function (pdf), is updated when observations, including their errors described by the likelihood, become available. Using Bayes' Theorem the prior pdf and the likelihood are multiplied to form the posterior pdf, which is the solution to the full data assimilation problem. So, data assimilation is more general than synchronization.

This relation between the two becomes apparent when the data assimilation is assumed to be linear, revealing a close connection between synchronization and the Kalman Filter. The main difference is that synchronization uses a tunable parameter to set the strength of the relaxation term, while in the Kalman Filter the Kalman gain is determined completely by the error covariances of model and observations. As is well known, the Kalman Filter provides the optimal gain, in the sense of minimal unbiased posterior errors, so the Kalman Filter informs us how to choose the tuning parameter in synchronization. The same is true when we use time embedding, in which the Kalman Smoother provides the optimal gain, in a linear system. It should also be mentioned that in synchronization, observations are used several times in the time‐embedding framework, which is possible to include in a Kalman Smoother, but the formulation would become rather complicated as correlations between model and observation errors would have to be taken into account. Synchronization typically does not worry about these correlations, as observation errors are assumed to be negligible.

When the system is nonlinear, synchronization could be used, indirectly, in a proposal density in nonlinear data assimilation. As pointed out by e.g. van Leeuwen (1999), the proposal density allows for enormous freedom in changing the model equations, indeed allowing for extra terms with tunable parameters. For instance van Leeuwen (1999) suggests the use of an Ensemble Kalman Filter as proposal in a particle filter, in which the observational‐error covariances are made much smaller than the true observational errors. Furthermore, observations can be used several times without complicating the algorithm *p* *e* *r* *s* *e*.

With this proposal density freedom in mind, we extend the synchronization method of Rey *et al.* (2014a, 2014b) to high‐dimensional settings, in which observation errors are non‐negligible. To this end, we introduce ensemble methods to avoid the propagation of very large matrices by the linearized model, and we make the method more robust to observational noise by observing a larger proportion of the system.

In section 2 we describe the methodology used to construct both the synchronization and the ensemble‐based synchronization frameworks and explore the similarities of the latter with the ensemble smoother. In section 3 we present results on both frameworks using the Lorenz'96 model in up to 1000‐dimensional settings. In section 4, we summarize and discuss our results and present conclusions.

## 2 Methodology

### 2.1 *Synchronization*

The initial stage of this work follows the synchronization ideas of Rey *et al.* (2014a, 2014b). The method explores the use of time‐advanced embeddings, to bring additional information from measurements ahead back to the present time. The main idea is that, after stabilizing the synchronization manifold, very precise estimates of unobserved variables are generated and these estimates allow an accurate prediction of the variables, over a significant forecast period. The effect of measurement noise in this type of system is also tested. Previous experiments with observation noise in lower‐dimension systems were performed by Rey *et al.* (2014b), showing that, as noise levels increase, the estimate's accuracy degrades significantly.

The synchronization framework can be summarized as follows. Define the state $\text{x}\in {ℜ}^{D_{x}}$ and observations $\text{y}\in {ℜ}^{D_{y}}$ at each time step of the model:

(1) $$\frac{d\text{x}(t)}{dt}=f\{\text{x}(t)\},$$

where *f*{**x**(*t*)} is the nonlinear model.

- Define *D*_d_ as the delay dimension, containing the time embeddings to include additional information from *D*_*y*_ measurements at different times in a time interval [*t*,*t*+(*D*_d_−1)*τ*], where *τ* is a constant time interval. Note the similarity with a fixed‐lag smoother.
- In the embedding dimension *D*_e_=*D*_d_∗*D*_*y*_, construct vectors $\text{S}\in {ℜ}^{D_{e}}$ related to the states and $\text{Y}\in {ℜ}^{D_{e}}$, related to the observations, as:
  (2) $$\begin{array}{ll} \text{S}\{\text{x}(t)\}= & ({\left[H\{\text{x}(t)\}\right]}^{T},{\left[H\{\text{x}(t+\tau )\}\right]}^{T},\\ & \;\;...,\left[H\{\text{x}(t+(D_{d}-1)\tau )\}\right]{}^{T}){}^{T}\; \end{array}$$
  and
  (3) $${\text{Y}(t)=\{\text{y}{\left(t\right)}^{T},\text{y}{\left(t+\tau \right)}^{T},...,\text{y}{\left(t+(D_{d}-1)\tau \right)}^{T}\}}^{T},$$
  in which **S**(**x**) is a map from physical to an embedding space and *H*(**x**) is the observation operator at each observation time, a map ${ℜ}^{D_{x}}\to {ℜ}^{D_{y}}$, which can be linear or nonlinear. For simplicity, we assume that *H* is the same at each time instant, but it is straightforward to make *H* time dependent.
- Calculate the Jacobian matrix *∂* **S**{**x**(*t*)}/*∂* **x**(*t*). To this end, we need to evaluate
  (4) $$\frac{\partial H\{\text{x}(t+\tau )\}}{\partial \text{x}(t)}=\text{H}\text{F}{\left(\text{x}\right)}_{0\to \tau },$$
  in which **H** is the Jacobian of the map *H* and **F**(**x**)_0→*τ*_ is the linearized model, so the Jacobian of the nonlinear model, from time 0 to *τ*. Then, this Jacobian matrix *∂* **S**{**x**(*t*)}/*∂* **x**(*t*) is of size *D*_e_×*D*_*x*_, where *D*_*x*_ is the number of dimensions of the system.
- Calculate the pseudoinverse *∂* **S**{**x**(*t*)}/*∂* **x**(*t*)^*†*^ of the Jacobian matrix, using a singular value decomposition (SVD). Since *∂* **S**{**x**(*t*)}/*∂* **x**(*t*) is a *D*_e_×*D*_*x*_ matrix, this can be prohibitively expensive for high‐dimensional systems.
- Finally, calculate the variable evolution with time:
  (5) $$\frac{d\text{x}(t)}{dt}=f\{\text{x}(t)\}+g{\frac{\partial \text{S}\{\text{x}(t)\}}{\partial \text{x}(t)}}^{†}\{\text{Y}(t)-\text{S}(t)\},$$
  where *g* is a coupling constant, which is a tuning parameter in synchronization.
- Keep iterating the whole process (stages (i)–(v)) from time *t*+1 on, until the last available observation.

The pseudoinverse *∂* **S**{**x**(*t*)}/*∂* **x**(*t*)^*†*^ spreads the information from the observed variables at measurement times to all unobserved variables of the model, at time *t*. It is important to note that not all observations that are available in the time interval [*t*,*t*+(*D*_d_−1)*τ*] are used, but only those *τ* time steps apart. The idea is that observations in between do not carry much new information, thus reducing the computational effort. Also note that one has to recalculate **S**(**x**) at every time step, and hence linearize the model at every time step over the time interval [*t*,*t*+(*D*_d_−1)*τ*]. This, again, is extremely expensive for larger models.

### 2.2 *The ensemble‐based synchronization*

The construction of the Jacobian matrix in the synchronization formulation requires the propagation forward in time of a *D*_*x*_×*D*_*x*_ matrix. In higher‐dimension systems, this may bring computational and storage issues. Instead, we will explore an ensemble framework here, similar to an Ensemble (Kalman) Smoother (van Leeuwen and Evensen, 1996; van Leeuwen, 1999; Evensen and van Leeuwen, 2000).

The ensemble methodology can be described by the following steps (the Appendix A gives the pseudocode):

- At time *t*=0, generate an ensemble of *N*_ens_ initial members by randomly perturbing **x**(0), i.e. members are isotropically distributed around **x**(0).
- Take the mean of these perturbed states:
  (6) $$\bar{\text{x}}(0)=\frac{1}{N_{\text{ens}}}\sum_{i=1}^{N_{\text{ens}}}{\text{x}}_{i}(0)$$
  and the difference between each member and the mean:
  (7) $$X_{i}(0)={\text{x}}_{i}(0)-\bar{\text{x}}(0),$$
  in which ***X***_*i*_(0) is the *i*th column of the initial ensemble perturbation matrix ***X***(0), a *D*_*x*_×*N*_ens_ matrix, and *N*_ens_ is the number of ensemble states or members.
- Propagate forward in time each full ensemble member for *τ* time steps, where *τ* is a chosen constant time interval, and form the ensemble perturbation matrix ***X***(*τ*) with the same dimensions as ***X***(0). Repeat this process for (*D*_d_−1) times to cover the full time window [*t*,*t*+(*D*_d_−1)*τ*] with the ensemble.
- Generate the augmented *D*_*e*_‐dimensional vectors **S** and **Y**, such as in equations (2) and (3), noting that the states **x** in **S**{**x**(*t*)} are the ensemble means at each embedding time.
- To calculate *∂* **S**(**x**(*t*))/*∂* **x**(*t*) we note that, approximately:
  (8) $$H\{X(\tau )\}\approx \text{H}F{\left(\text{x}\right)}_{0\to \tau }X(0),$$
  where again **H** is the Jacobian of the map *H* and **F**(**x**)_0→*τ*_ is the linearized model, so the Jacobian of the nonlinear model, from time 0 to *τ*. Note that in the nonlinear case, *H*{***X***(*τ*)} is defined by:
  (9) $$\begin{array}{ll} H\{X(\tau )\}= & [H\{{\text{x}}_{1}(\tau )\}-\bar{H\{\text{x}(\tau )\}},\\ & \;\;\text{⋯}\;,H\{{\text{x}}_{N_{\text{ens}}}(\tau )\}-\bar{H\{\text{x}(\tau )\}}]\; \end{array}$$
  in which
  (10) $$\bar{H\{\text{x}(\tau )\}}=\frac{1}{N_{\text{ens}}}\sum_{i=1}^{N_{\text{ens}}}H\{{\text{x}}_{i}(\tau )\}.$$
  This allows us to approximately compute the Jacobian ***F***(**x**)_0→*τ*_ as:
  (11) $$\text{H}F{\left(\text{x}\right)}_{0\to \tau }\approx H\{X(\tau )\}{\left\{X(0)\right\}}^{†},$$
  where {***X***(0)}^*†*^ is the pseudoinverse of ***X***(0). Note that we do not need to calculate this pseudoinverse explicitly (see below). The full Jacobian matrix *∂* **S**(**x**(*t*))/*∂* **x**(*t*) can be constructed as:
  $$\frac{\partial \text{S}\{\text{x}(t)\}}{\partial \text{x}(t)}=\left(\begin{array}{c} \text{H(x)}\\ \text{H}F{\left(\text{x}\right)}_{0\to \tau }\\ \vdots \\ \text{H}F{\left(\text{x}\right)}_{0\to (D_{d}-1)\tau } \end{array}\right)$$
  which can now be rewritten as:
  $$\frac{\partial \text{S}\{\text{x}(t)\}}{\partial \text{x}(t)}\approx \left(\begin{array}{c} H\{X(0)\}{\left\{X(0)\right\}}^{†}\\ H\{X(\tau )\}{\left\{X(0)\right\}}^{†}\\ \vdots \\ H\{X((D_{d}-1)\tau )\}{\left\{X(0)\right\}}^{†} \end{array}\right)$$
  So we can now calculate the pseudoinverse of *∂* **S**{**x**(*t*)}/*∂* **x**(*t*) by noting that each sub‐matrix above has the factor {***X***(0)}^*†*^ in common, as:
  $${\frac{\partial \text{S}\{\text{x}(t)\}}{\partial \text{x}(t)}}^{†}\approx X(0){\left(\begin{array}{c} H\{X(0)\}\\ H\{X(\tau )\}\\ \vdots \\ H\{X((D_{d}-1)\tau )\} \end{array}\right)}^{†}$$
  This shows that we need to compute the pseudoinverse of a *D*_e_×*N*_ens_ measured ensemble perturbation matrix, which we can do via truncated SVD.
- Use the coupled dynamics to propagate the model states forward in time, as in Eq. (5).
- Keep iterating the whole process (stages (i)–(vi)) from time *t*+1 on, until the last available observation, at time *t*+(*D*_d_−1)*τ*.

In our implementation, we take the coupling constant *g* which appears in Eq. (5) equal to 1. Also, the ensemble version of this synchronization scheme utilizes a localization method to reduce the influence of observations which are far away from the variables (Houtekamer and Mitchell, 1998); Asch *et al.* (2016) give an overview on localization. We assume that we can attribute a position in physical space to each observation (location on the Lorenz ring). The implementation of localization is as follows:

- For each variable, calculate the distance *d* (along the ring) between the position in physical space and all the existing observations in the system.
- Define a threshold *l* *o* *c* for this distance. If this limit is exceeded, discard the influence of the observation in the variable. Otherwise, calculate the influence for each variable:
  (12) $$iloc=\exp\left[-\{d^{2}/(2loc^{2})\}\right]$$
  storing each value in a vector **iloc**.
- Compute the Schur product between **iloc** and the difference (**Y**(*t*)−**S**(*t*)) to generate a new variable evolution from Eq. (5):
  (13) $$\frac{d\text{x}(t)}{dt}=f\{\text{x}(t)\}+g{\frac{\partial \text{S}\{\text{x}(t)\}}{\partial \text{x}(t)}}^{†}[\text{iloc}\circ \{\text{Y}(t)-\text{S}(t)\}].$$

Finally, steps (v) and (vi) are not the most efficient way to perform the necessary calculations. Instead, it would be more efficient to first calculate

(14) $$\begin{array}{ll} & [([H\{X(0)\}],[H\{X(\tau )\}],\\ & \;\;\text{⋯}\;,[H\{X((D_{d}-1)\tau )\}]){}^{T}]{}^{†}[\text{iloc}\circ (\text{Y}-\text{S})], \end{array}$$

which results in a vector of size *N*_ens_, which is then used to right‐multiply ***X***(0). This is particularly more efficient in higher‐dimensional cases.

The methodology described above has strong similarities with an Ensemble Smoother (van Leeuwen and Evensen, 1996). The Ensemble Smoother update can be formulated as:

(15) $$\begin{array}{ll} {\text{x}}^{a} & ={\text{x}}^{f}\;\\ & +X{\left\{H(X)\right\}}^{T}(H(X)\left\{H(X)\right\}{}^{T}+\text{R}){}^{-1}\{\text{Y}(t)-\text{S}(t)\}.\; \end{array}$$

The main differences between Ensemble Smoothers and our Ensemble Synchronization are the following. Synchronization ignores observation errors in the gain. This allows for an inversion of *H*(***X***) directly, instead of (*H*(***X***){*H*(***X***)}^T^+**R**). The latter is a larger matrix, but, of course, in efficient implementations the matrix to be inverted in an ensemble smoother is of size *N*_ens_×*N*_ens_. Using localization, the matrices in both methods are of similar size. Ignoring observation errors is perhaps a weakness, but it is partly compensated for by the extra tuning possibility via the factor *g*. Another difference is that observations are used multiple times in time‐embedded synchronization. Using the same observations multiple times in ensemble smoothers would introduce correlations between the errors of the state iterates and the observation errors, complicating the scheme considerably. Synchronization is not hampered by that issue, and observations are used several times to increase the observability of the system. This is a crucial advantage of synchronization.

This comparison also holds true for Ensemble Synchronization and variational methods which employ ensembles to avoid adjoint calculations, like 4DEnsVar (e.g. Liu *et al.*, 2008; Fairbairn *et al.*, 2014; Gustafsson and Bojarova, 2014), and iterative Ensemble Smoothers like the IEnKS of Bocquet and Sakov (2014), as these methods are all based on the same assumptions and use the same ensemble space–time correlations as ensemble smoothers. The IEnKS, differently from the one presented in this work, reuses all the observations inside the data assimilation window, whereas our scheme uses only the observations at every *τ* time steps in the [*t*,*t*+(*D*_d_−1)*τ*] window. The scheme does use observations multiple times, but with inflated covariances. Their approach factorizes the likelihood and assimilates the resulting sequence of likelihoods sequentially. It is important to recall that our methodology targets a different purpose. While data assimilation methods aim to approximate the true posterior pdf, this synchronization technique tries to find a model trajectory that follows the observations as close as possible, to synchronize with the true trajectory. Therefore, as mentioned in the introduction, synchronization is not a complete data assimilation method, as uncertainties in model and observations are not incorporated explicitly in the formulation. A very efficient use we see for the synchronization scheme is as part of a proposal density in a more comprehensive data assimilation method, like a particle filter. As shown by e.g. van Leeuwen (2009), observations can be used as often as one would wish in a proposal.

## 3 Experiments and results

The metric typically used in synchronization to monitor the synchronization error (SE) is the root mean square error (RMSE):

(16) $$\mathrm{SE}(t)=\sqrt{\frac{1}{{}_{\mathrm{Dx}}}\sum_{k=1}^{{}_{\mathrm{Dx}}}{\left\{x_{\text{true}}^{k}(t)-x^{k}(t)\right\}}^{2}}.$$

In this work, ‘*x*_true_’ is considered in Eq. (16), as we performed twin experiments, in which the truth is artificially generated. In real cases, however, knowledge about the truth relies on the observations and so ‘*x*_true_’ would be substituted by ‘*y*’ and *x* ^*k*^ should be replaced by *H*(*x*) in this evaluation metric.

Note that, in all cases described below, we have varied the initial conditions and random number realizations, all leading to similar qualitative and quantitative results. Hence, these results can be considered typical for the behaviour of the system at hand.

### 3.1 *Synchronization results using matrix propagation*

We performed twin experiments using a *D*_*x*_‐dimensional Lorenz'96 model,

(17) $$\frac{dx_{a}}{dt}=(x_{a+1}-x_{a-2})x_{a-1}-x_{a}+f$$

for *a*=1,...,*D*_*x*_ and a forcing parameter *f*=8.17, which guarantees chaotic behaviour for *D*_*x*_=20, 100 and 1000. A fourth‐order Runge–Kutta scheme was applied, with Δ*t* = 0.01 and a constant time interval *τ*=10Δ*t*. An observation noise sampled from a normal distribution with standard deviation *σ*_o_ = 0.1 was used.

The coupling constant *g* in Eq. (5) can be equal to 1 and we still obtain synchronization. However, using *g*=0.1, and therefore reducing the influence of the whole coupling term in Eq. (5) by a factor of 10, produces slightly better results. Therefore, we use *g*=0.1 in all synchronization experiments. After some experimentation, we have decided to use 2*D*_*y*_ singular values in the computation of the SVD for the pseudoinverse of the Jacobian matrix, as this produced the most stable results. We choose this value for all the cases shown in this subsection.

#### 3.1.1 *20‐variable case*

In this case, five variables were observed equidistantly on the Lorenz'96 ring, being used at every *τ* time steps, during a total length of the experiment of 10 000 time steps (comparable with Rey *et al.*, 2014a, 2014b).

**Figure 1:**
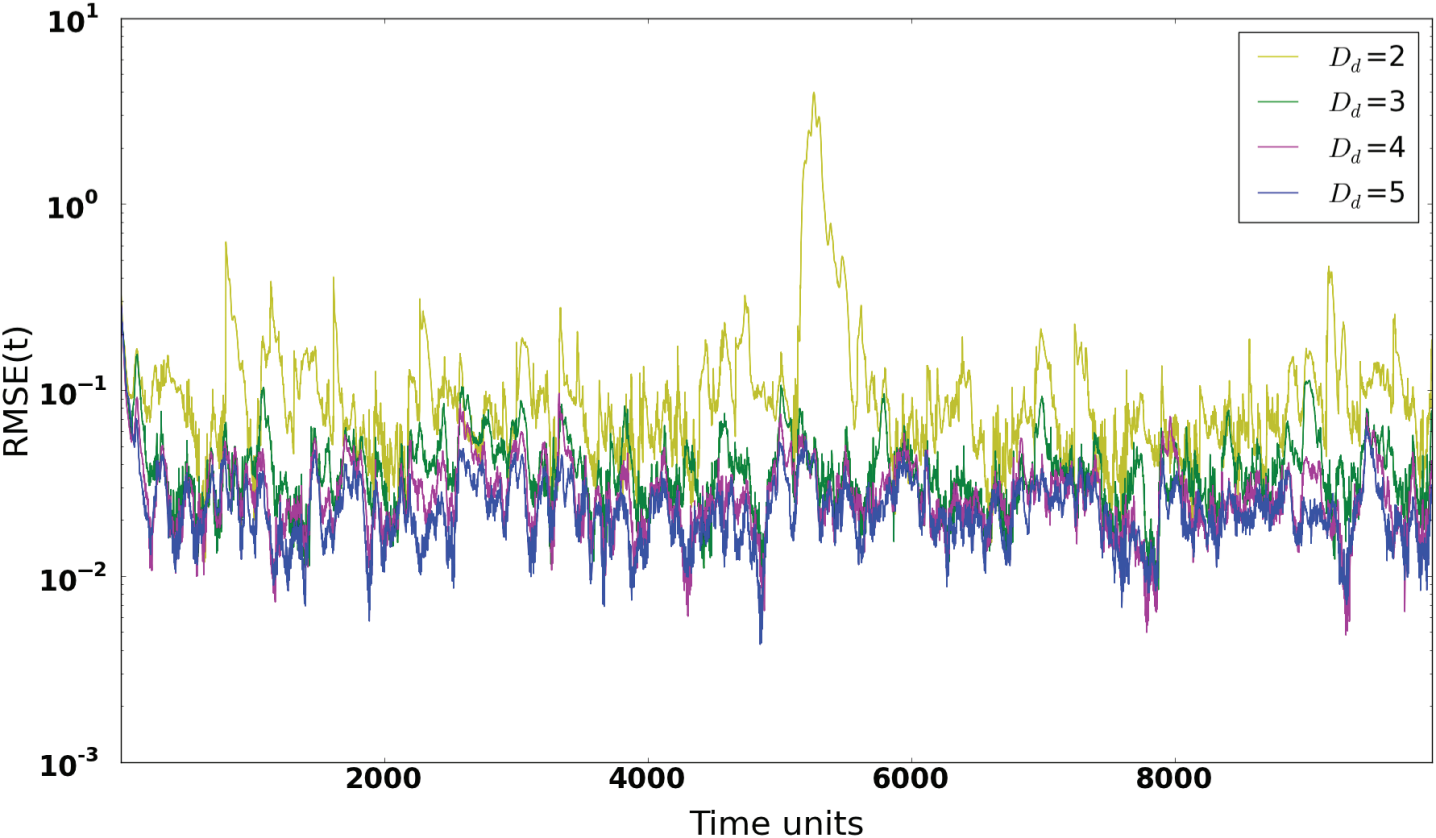
Synchronization Error (RMSE) for different delay dimensions in a 20‐variable system with five measured variables sampled equidistantly on the Lorenz'96 ring, using synchronization with matrix propagation. [Colour figure can be viewed at http://wileyonlinelibrary.com].

The size of the delay dimension *D*_d_ is a crucial factor, as it needs to be large enough to provide useful and additional information to compensate the lack of measurements, but small enough to keep the numerical stability of the pseudoinverse computation. Figure 1 shows the synchronization errors (RMSE) for different embedding intervals. The first noticeable point in the figure is the magnitude of the synchronization error (RMSE), compared to Rey *et al.* (2014a). While those authors have reached values of around 10^−15^, our results range between the order of 10^−2^ and 10^−3^. This is directly related to the inclusion of observational noise. Therefore, in our tests we define the system as synchronized when *R* *M* *S* *E*<*σ*_o_ and stabilized when no sharp peaks in the synchronization error are found along the run. Figure 1 illustrates the following:(i) an embedding interval of at least *D*_d_=3 is needed to reach RMSE values <*σ*_o_ and achieve synchronization;(ii) for *D*_d_=2, the system does not stabilize and shows moments of desynchronization;(iii) for *D*_d_=4 and *D*_d_=5, low RMSE values are obtained and convergence seems to have been reached.

**Figure 2:**
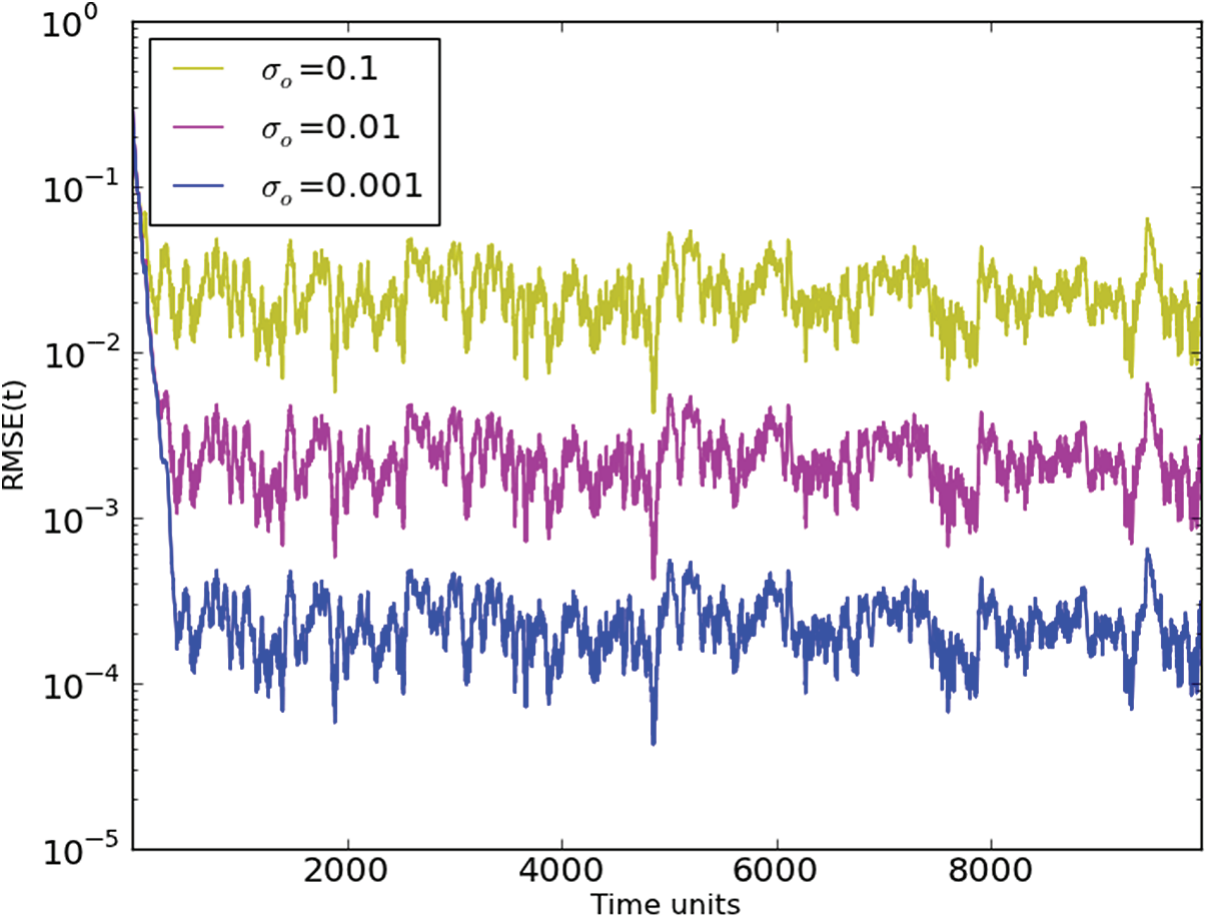
Synchronization Error (RMSE) for different standard deviations (σ) of observation noise in a 20‐variable system with five measured variables sampled equidistantly on the Lorenz'96 ring (D_d_=5), using synchronization with matrix propagation. [Colour figure can be viewed at http://wileyonlinelibrary.com].

In order to test the impact of the observation noise in the system, we varied *σ*_o_ between 0.1 and 0.001, as shown in Figure 2. It is clear that the measurement noise affects the quality of synchronization, particularly in the first 500 time steps. We used the same realizations for the observation noise in all experiments, and the similar time series for different values of *σ*_o_ show that we have reached synchronization in the linear regime. In general, we find *R* *M* *S* *E*≈(1/5)*σ*_o_.

**Figure 3:**
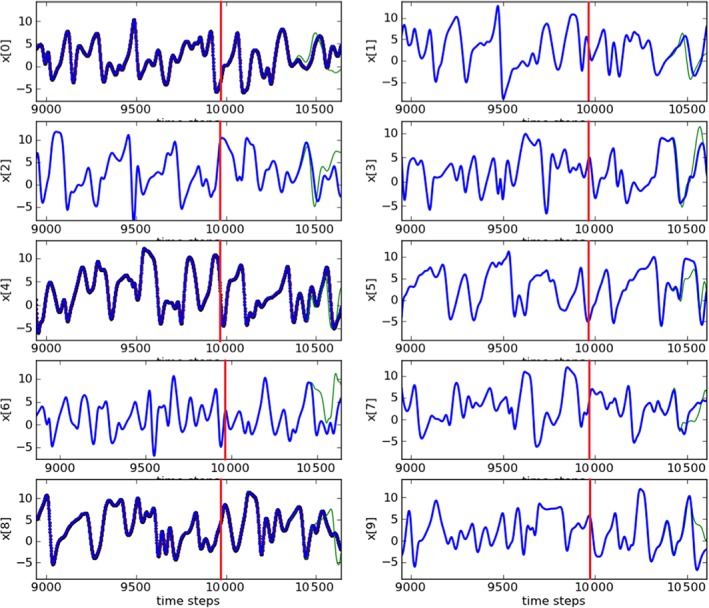
Trajectories of the first ten variables (y axes) and our estimates (upto 9960 time steps–red lines) and predictions (after 9960 time steps). The blue lines are the truth and the green lines are the estimates/predictions. The observed variables are: 0, 4 and 8. Synchronization with matrix propagation was used. [Colour figure can be viewed at http://wileyonlinelibrary.com].

**Figure 4:**
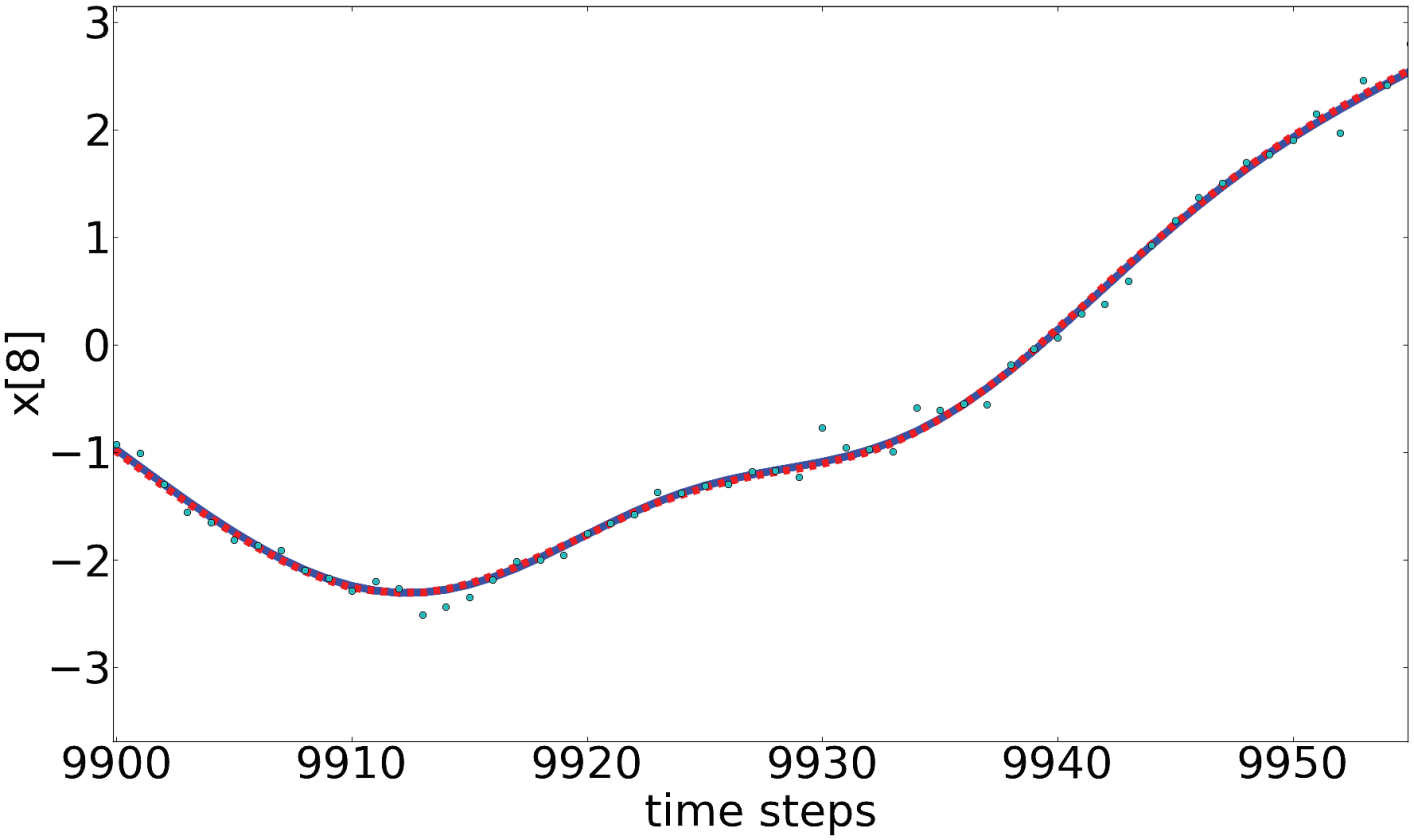
Trajectory of one of the variables in Figure 3, enlarged at the end of the estimation period. The blue line is the truth, the red dashed line is the estimate and the green dots are the observations. Synchronization with matrix propagation was used. [Colour figure can be viewed at http://wileyonlinelibrary.com].

After 9960 time steps (10 000 time steps minus (*D*_d_−1)*τ*, for *D*_d_=5), the system is set to run freely, i.e. no synchronization scheme is used. Figure 3 shows estimations and predictions for the first ten variables (and Figure 4 shows an expansion at the end of the estimation period of one of the variables to highlight the differences between the truth and the observations). During the estimation period, the green lines (estimates) match visually perfectly to the blue lines (truth). This shows how close our estimates are to the true values. Also, during prediction stage, very precise values are reached for almost 500 time steps and after that, predictions start to diverge from the truth, given the chaotic nature of the model. This prediction range is reduced compared to Rey *et al.* (2014a, 2014b), again related to the inclusion of observational errors.

**Figure 5:**
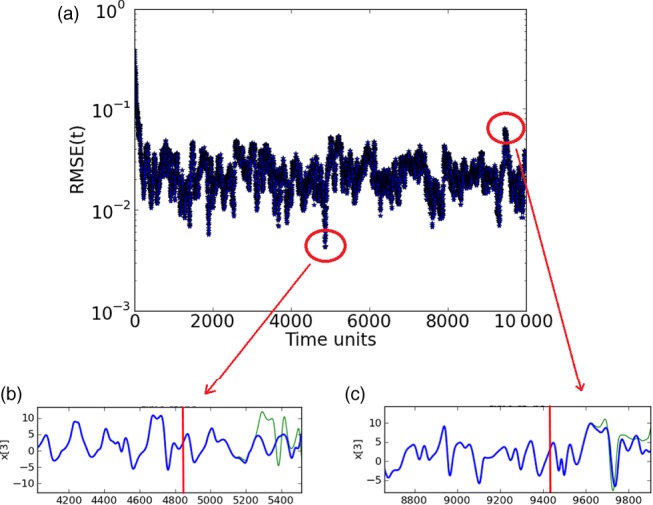
(a) Synchronization error (RMSE) for a 20‐variable system with five measured variables sampled equidistantly on the Lorenz'96 ring (D_d_=5), and unobserved variable estimates/predictions for estimation periods of (b) 4855 time steps and (c) 9454 time steps. The prediction range in (b) is of ≈400 time steps, while (c) is of ≈150 time steps. Synchronization with matrix propagation was used. [Colour figure can be viewed at http://wileyonlinelibrary.com].

The prediction range depends on how well synchronized the system is at the starting point of the prediction. Figure 5 shows that if, for instance, we start prediction at time step 4855, where we find minimal RMSE values, it is possible to predict an unobserved variable very precisely for around 400 time steps. If, however, we start predicting at time step 9454, when the RMSE value points out a less synchronized moment of the system, the prediction range for the variable is reduced, reaching around 150 time steps.

We believe that there are two reasons for encountering a reduced prediction range:

- poor initial conditions due to poor synchronization, as mentioned before; and
- the fact that, even if a tiny amount of observation noise is included, simulations will (at the end of the synchronization period) end up in a different part on the chaotic attractor, where local divergence of neighbouring trajectories might be larger. To exclude this effect, one should average over many simulations with different initial conditions and/or many prediction attempts, at different times.

We were not able to use synchronization when only one variable is observed and observation errors are included. Even increasing *D*_d_ to up to 20 did not help. Our conclusion is that there is not enough information in the one noisy observation to synchronize the system. This has changed dramatically when we increased the number of observed variables per time step to 5, which means that about 25% of the system is observed. As soon as we found synchronization, we could reduce the time‐embedding window to 40 (so *D*_d_ = 5), compared to a larger embedding interval needed in Rey *et al.* (2014a). We come back to this important point in the concluding section.

#### 3.1.2 *100‐variable case*

In this case, 25 variables were observed at every *τ*=10 time steps. Noting that after the initial transient period, the length of the estimation period is not the crucial point in finding synchronization, we have reduced the length of these experiments to 1000 time steps.

**Figure 6:**
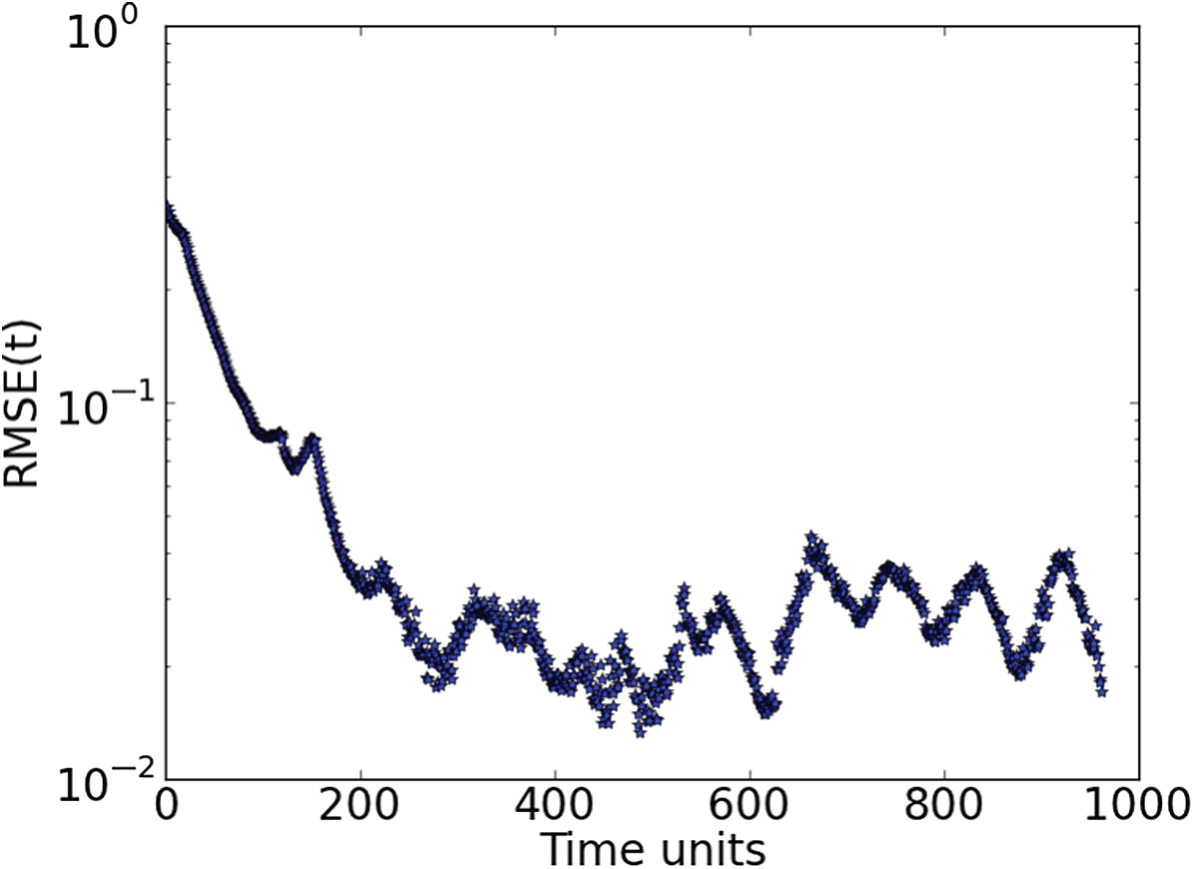
Synchronization error (RMSE) for a 100‐variable system with 25 measured variables sampled equidistantly on the Lorenz'96 ring (D_d_=5). Synchronization with matrix propagation was used. [Colour figure can be viewed at http://wileyonlinelibrary.com].

**Figure 7:**
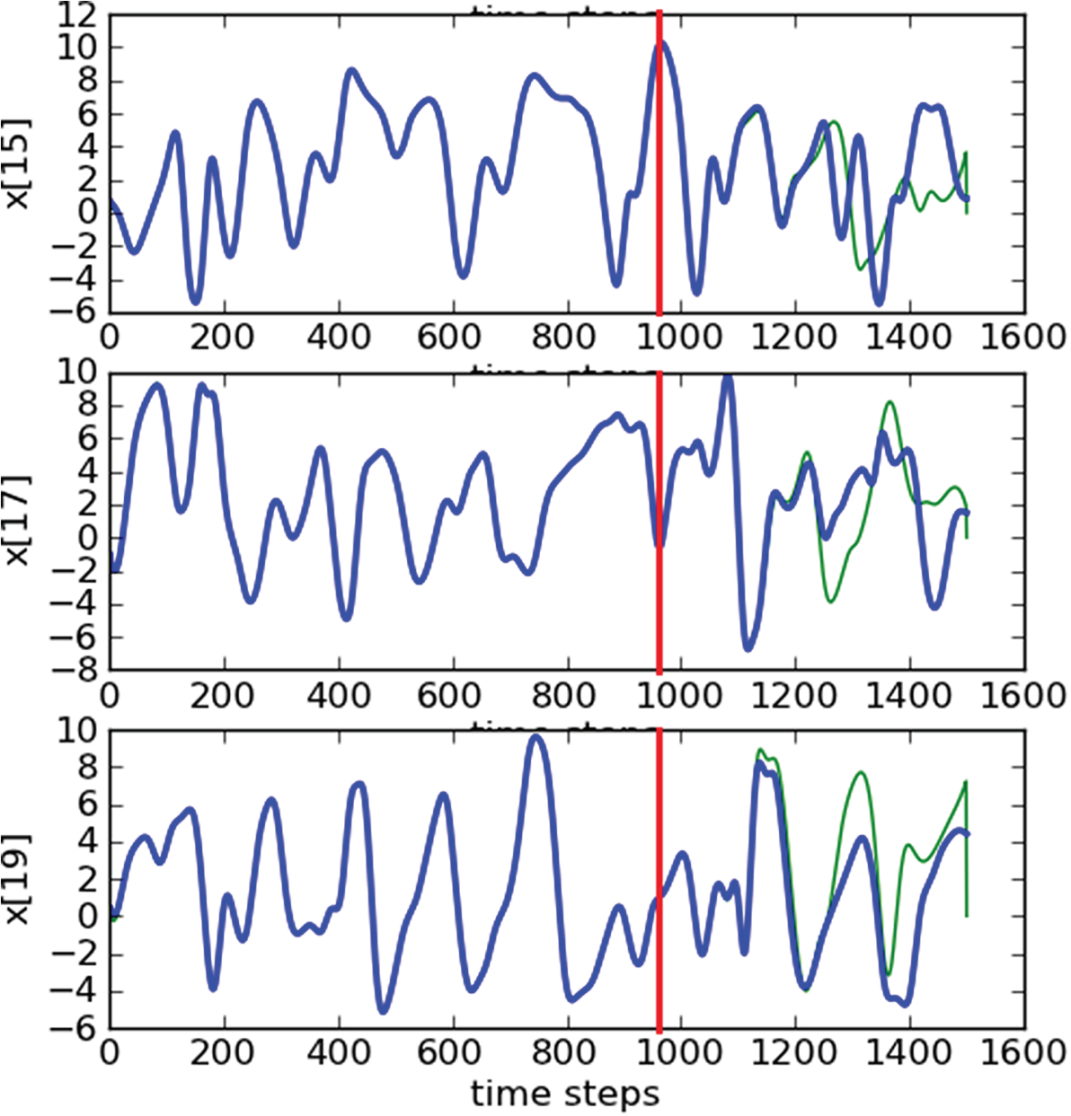
Trajectories of three unobserved variables in a 100‐dimension system. The blue lines are the truth and the green ones are the estimates. Predictions start at time step 960 (red lines). Synchronization with matrix propagation was used. [Colour figure can be viewed at http://wileyonlinelibrary.com].

Keeping the previous optimal value for the delay dimension *D*_d_=5, we show in Figure 6 the RMSE values decreasing to around 10^−2^, deriving good estimates of all variables throughout the estimation period, as shown for a few variables in Figure 7. Note that the estimation period in this case goes until time step 960, after which the prediction begins.

Accurate predictions are obtained for around 250 time steps. It is interesting to see that the dimension of the system has no influence on the synchronization error and the delay dimension.

#### 3.1.3 *1000‐variable case*

**Figure 8:**
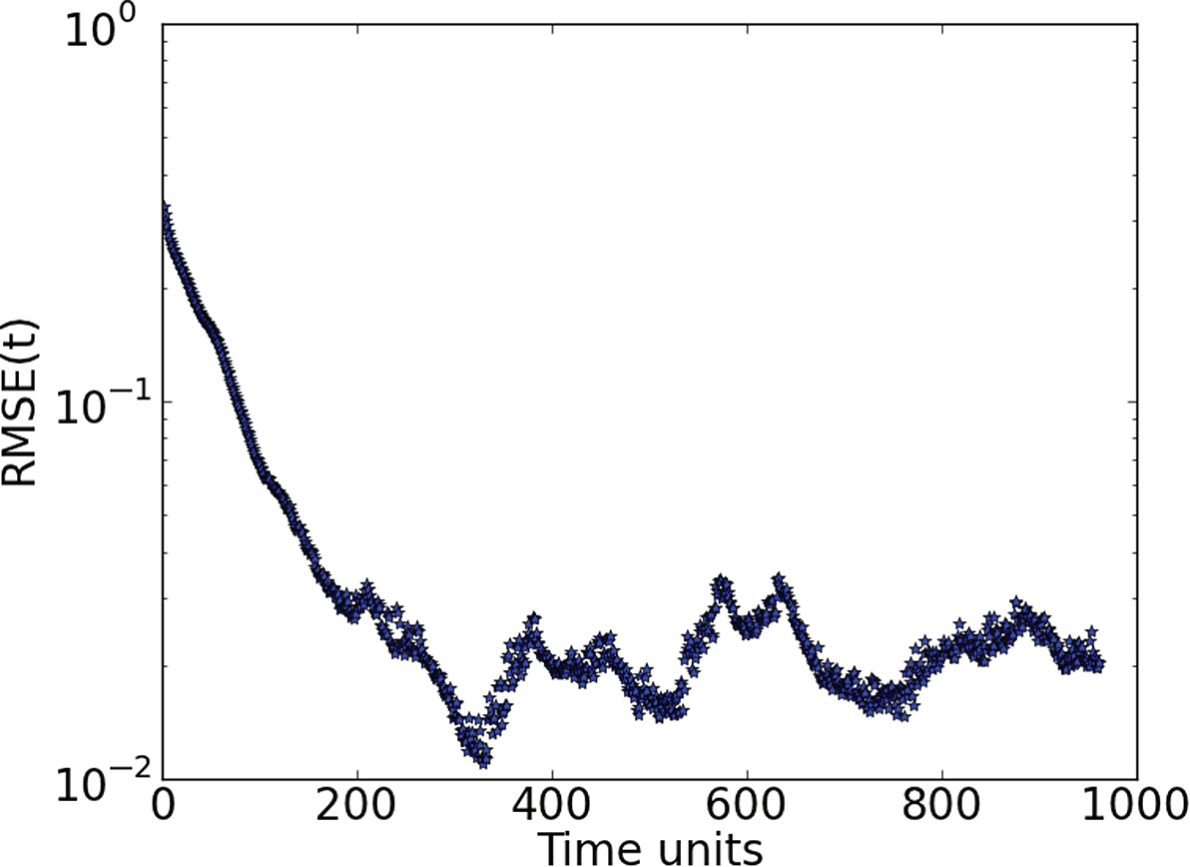
Synchronization error (RMSE) for a 1000‐variable system with 250 measured variables sampled equidistantly on the Lorenz'96 ring (D_d_=5). Synchronization with matrix propagation was used. [Colour figure can be viewed at http://wileyonlinelibrary.com].

**Figure 9:**
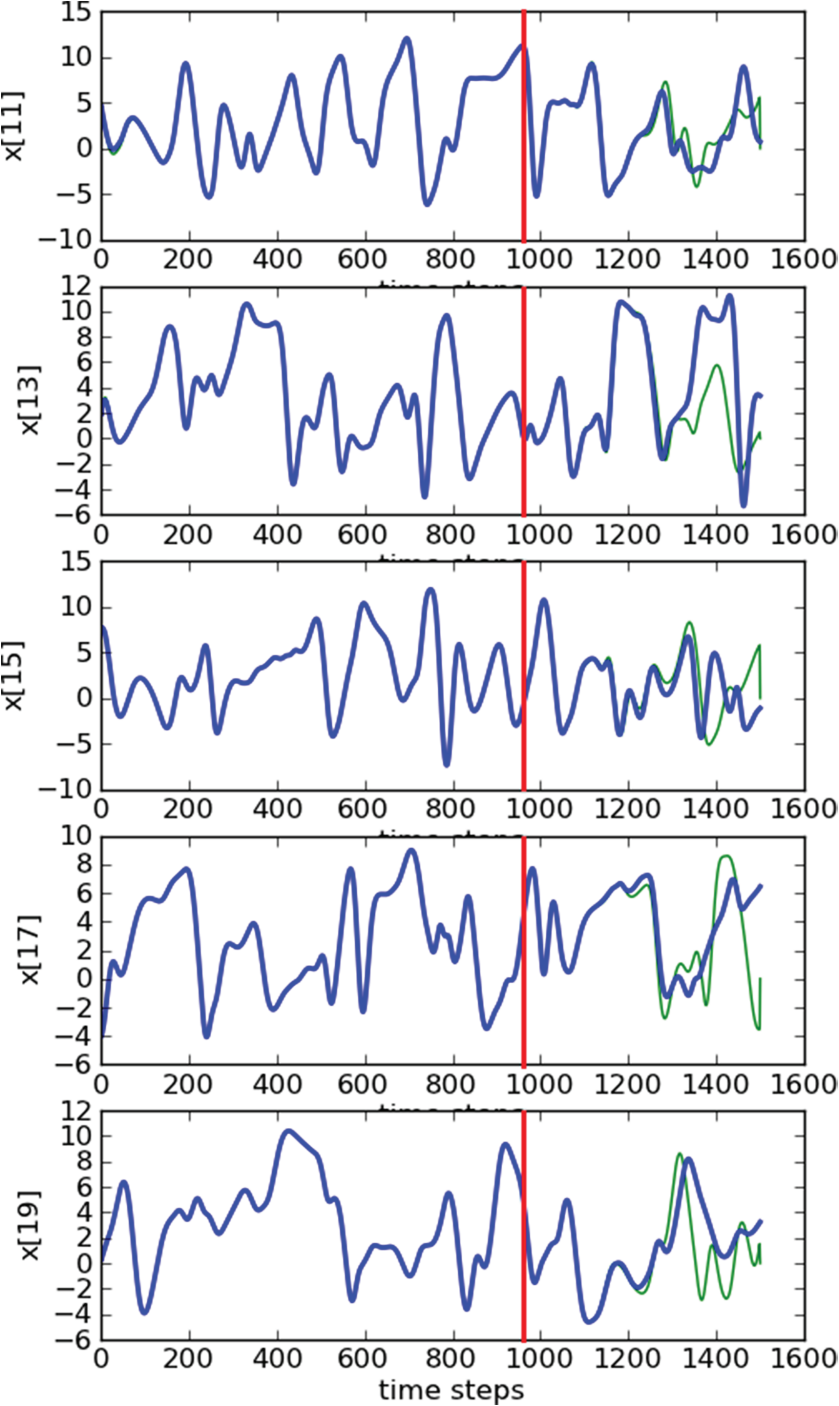
Trajectories of five unobserved variables in a 1000‐dimension system. The blue lines are the truth and the green ones are the estimates. Predictions start at time step 960 (red lines). Synchronization with matrix propagation was used. [Colour figure can be viewed at http://wileyonlinelibrary.com].

In this experiment, 250 variables were observed and the estimation period was also 960 time steps. Although the number of variables has increased, the delay dimension to compensate the lack of measurements is still the same, i.e. *D*_d_=5. Figure 8 shows synchronization with low RMSE values. Again, good estimates of all variables are produced as Figure 9 shows. Also, very precise predictions are obtained for more than 250 time steps, as is shown in Figure 9. Again, comparing with the 20‐ and 100‐dimensional cases, we find no dependence on the system size.

It should be mentioned that this run is extremely expensive, propagating a 1000 by 1000 matrix for 40 time steps at each time step. This is the main motivation to turn to the ensemble framework.

### 3.2 *Ensemble‐based synchronization results*

The previous results were obtained by propagating a matrix of size *D*_*x*_×*D*_*x*_ for (*D*_d_−1)*τ* time steps, for each forward model time step. This is extremely demanding computationally. In the following, we explore ensemble methods, reducing the computation by a factor of *D*_*x*_/*N*_ens_.

For these experiments, we basically used the same configuration as before, using 20‐, 100‐ and 1000‐variable systems. Like in the previous experiments, the same proportion of the system is observed (25*%*), so that every fifth variable is measured. Tests were performed for different ensemble sizes, delay dimensions *D*_d_ and also standard deviations *σ*_o_ for the measurement noises. Ensemble members are slightly perturbed, with a normal distribution of *N*∼(0,0.01). Different ensemble sizes were tested: 5, 15, 20, 50, 100 and 200 members, but we will show results only for the minimal number of ensemble members needed in each case, corresponding to the cheapest configurations. The number of singular values used in the computation of the SVD is equal to the number of ensemble members used. This is valid for all the ensemble cases shown next.

#### 3.2.1 *20‐variable case*

For this case, again, five variables were observed, being used at every ten time steps *τ*, during 10 000 time steps.

As the main purpose of implementing an ensemble version of the synchronization system is to make it more computationally feasible for realistic systems, the first crucial information in these experiments is the number of ensemble members needed to achieve synchronized results. Tests were performed for different numbers of ensemble members and very precise estimations were obtained by using only five members in the ensemble.

**Figure 10:**
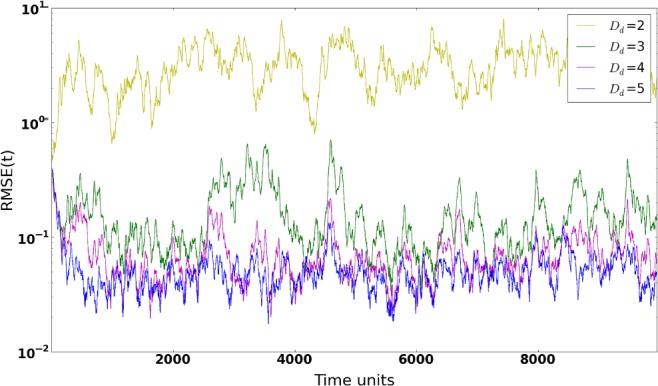
Synchronization error (RMSE) for different delay dimensions in a 20‐variable system with five measured variables sampled equidistantly on the Lorenz'96 ring and five ensemble members. Ensemble synchronization was used. [Colour figure can be viewed at http://wileyonlinelibrary.com].

Regarding the effective embedding interval to make the system stabilize and synchronize, Figure 10 shows the synchronization errors (RMSE) for different *D*_d_ values. In these results, we note that for *D*_d_=3, the system does not stabilize, showing peaks of less synchronized moments. For *D*_d_=4 and 5, low RMSE values are reached, however a more stable and synchronized system is obtained while using *D*_d_=5, which tends to stabilize faster and more consistently. Tests were also performed for delay dimensions varying from *D*_d_=6 to 12, showing synchronization in all of these cases. However, for *D*_d_=11 and 12 desynchronization is observed at some time steps along the run, although the mean RMSE values remain of the order of 10^−2^.

It is also noticeable that the synchronization scheme presented previously has slightly lower RMSEs, compared to the ensemble scheme, but the latter is still providing RMSE values well below the observation noise.

**Figure 11:**
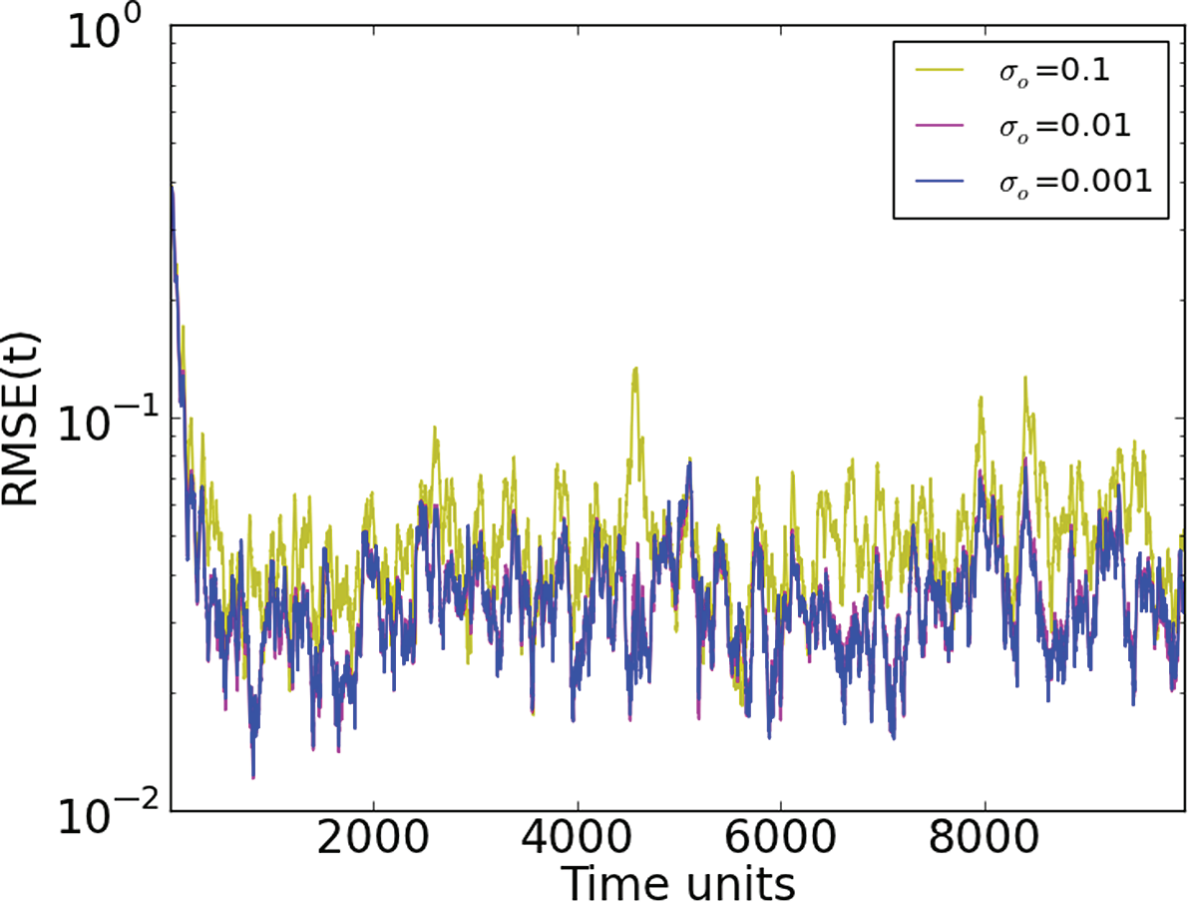
Synchronization error (RMSE) for different standard deviations (σ) of observation noise in a 20‐variable system with five measured variables sampled equidistantly on the Lorenz'96 ring and five ensemble members (D_d_=5). Ensemble synchronization was used. [Colour figure can be viewed at http://wileyonlinelibrary.com].

**Figure 12:**
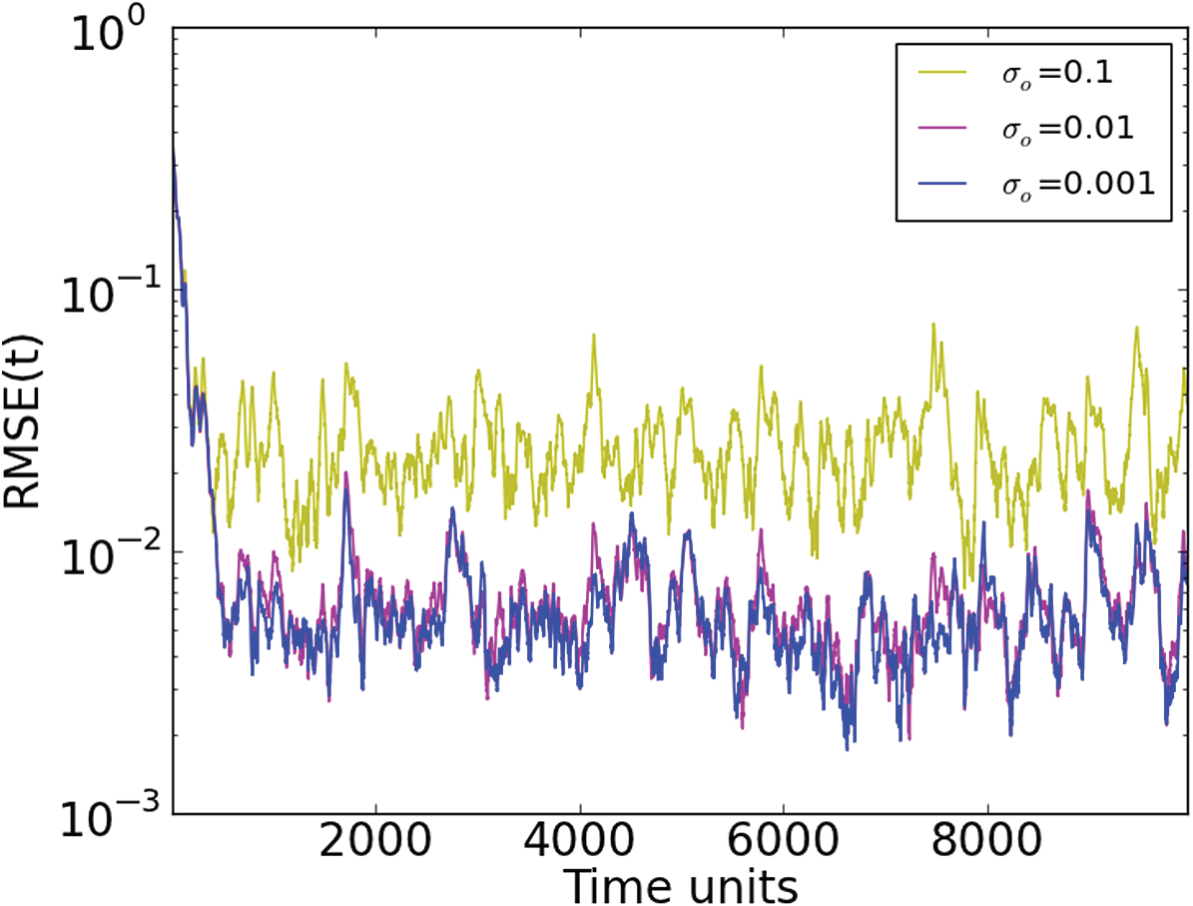
As Figure 11, but for 100 ensemble members. [Colour figure can be viewed at http://wileyonlinelibrary.com].

We have also tested the impact of the size of the observation noise in the ensemble system in combination with ensemble size. Figure 11 shows that, for five members, RMSE seems to stabilize at *σ*=0.01 level, even for *σ*_o_=0.001. This suggests that Monte‐Carlo noise is an important contributor to RMSE. To investigate this further, the same case was run with 100 ensemble members (Figure 12). As the number of members increases, the system tends to get more stabilized at *σ*=0.1, reaching smaller RMSE values for all observation noise levels, compared to the results in Figure 11.

**Figure 13:**
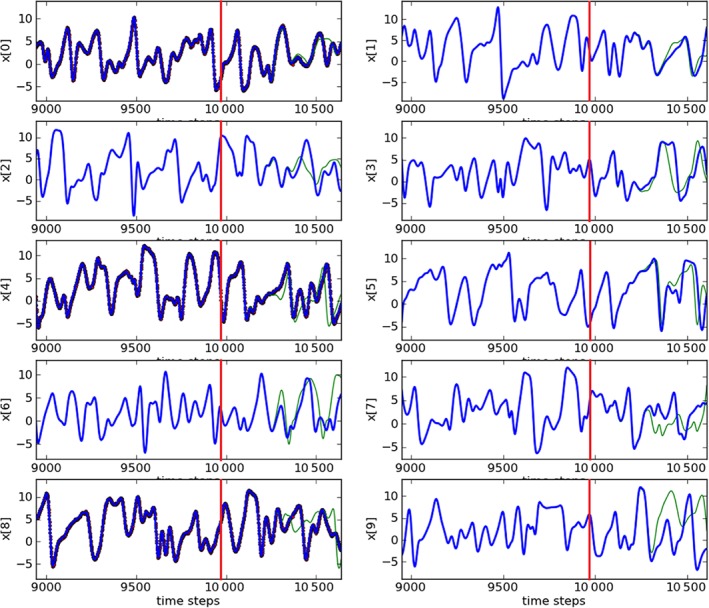
As Figure 3, but for the ensemble‐based synchronization system. [Colour figure can be viewed at http://wileyonlinelibrary.com].

The predictions for the first ten variables are shown in Figure 13. Also for this experiment, our estimates are very close to the truth. Although prediction ranges for this experiment are reduced compared to the ones for the synchronization scheme shown in Figure 3, very precise values are reached for around 300 time steps, which is still a considerable period.

#### 3.2.2 *100‐variable case*

**Figure 14:**
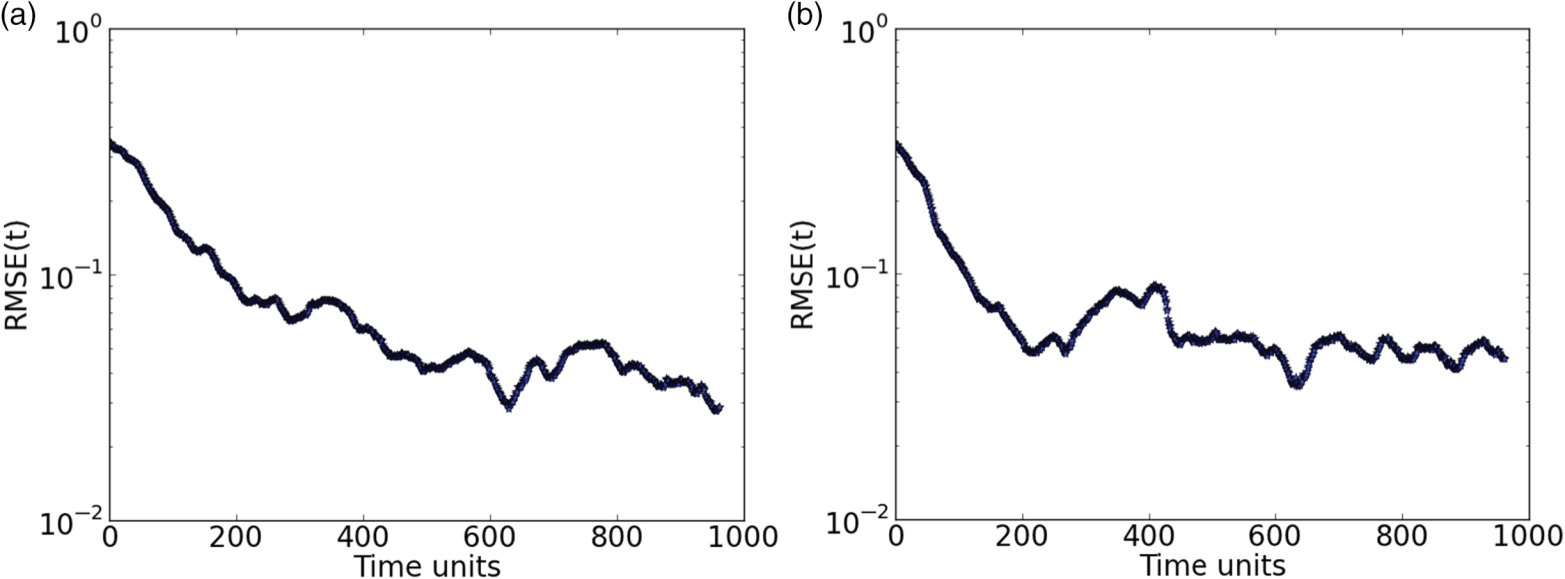
Synchronization error (RMSE) for a 100‐variable system with 25 measured variables sampled equidistantly on the Lorenz'96 ring (D_d_=5): (a) fifteen ensemble members, without localization, and (b) five ensemble members with localization applied. Ensemble synchronization was used. [Colour figure can be viewed at http://wileyonlinelibrary.com].

This case considers 25 observed variables, the same estimation period for the previous 100‐variable case, and a delay dimension of *D*_d_=5. The main difference from the 20‐variable case is that, due to the increase of number of variables to 100, in this experiment 15 ensemble members were required to stabilize the system and make the model synchronize with the truth. Figure 14(a) shows good results for this configuration, with RMSE values decreasing to a magnitude of 10^−2^.

**Figure 15:**
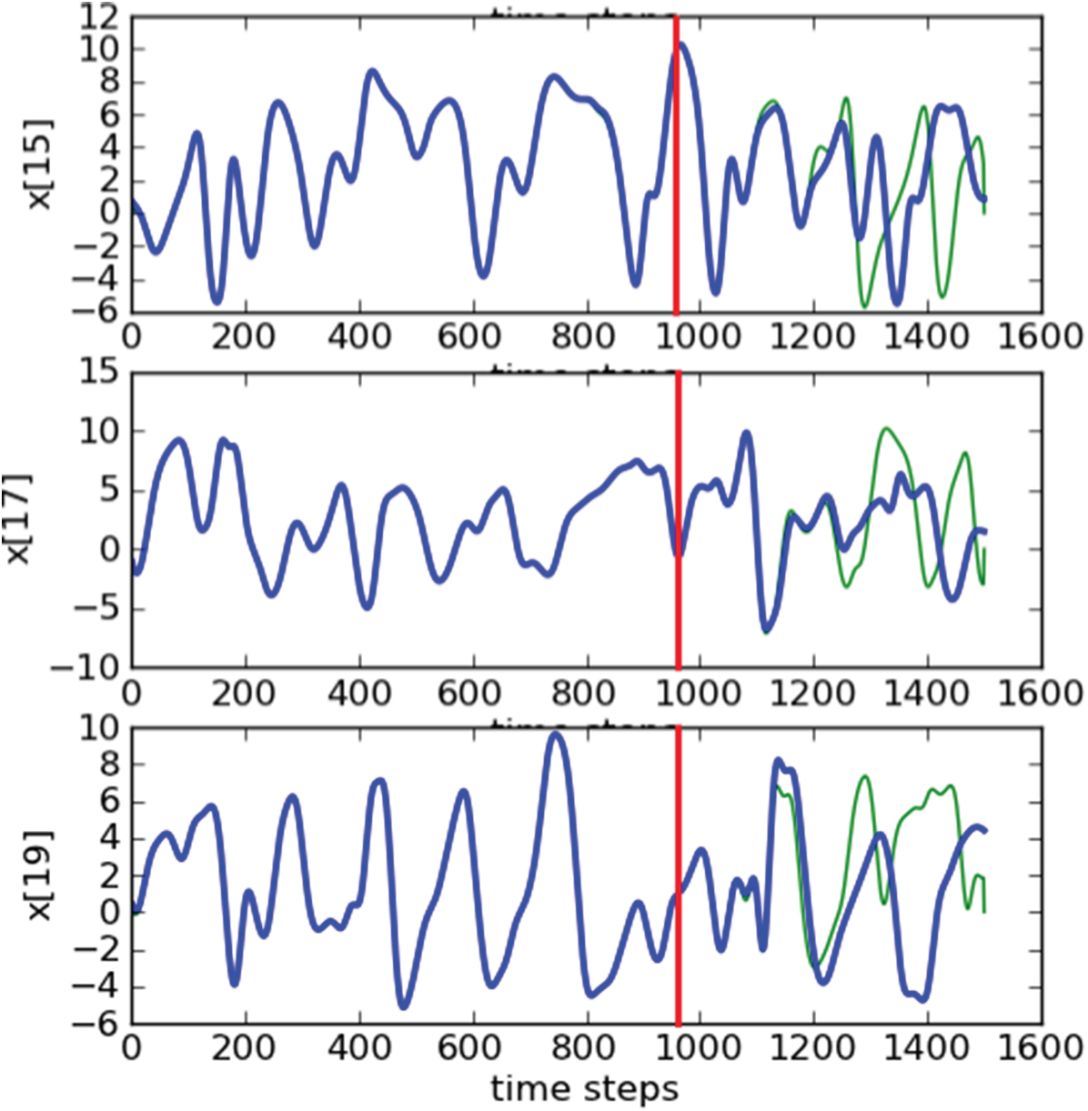
Trajectories of three unobserved variables in a 100‐dimension system for the ensemble‐based synchronization system with localization and five ensemble members. The blue lines are the truth and the green ones are the estimates. Predictions start at time step 960 (red lines). Ensemble synchronization was used. [Colour figure can be viewed at http://wileyonlinelibrary.com].

Aiming to construct a framework that can be used in high‐dimensional systems, the scheme requires the implementation of a localization method to reduce the influence of spurious correlations arising from using a small ensemble size. We use a localization radius of influence *r* *a* *d*=3, so that any observations located further than the threshold *l* *o* *c*=3×*r* *a* *d* are ignored by the variable. The localization function has an exponential shape. By localizing the effect of these distant observations in the variables, we were able to reduce the number of ensemble members from 15 to 5. Results (Figure 14(b)) show that, with localization, the system stabilizes and synchronizes faster than without localization. Estimates and predictions for some variables are shown in Figure 15.

#### 3.2.3 *1000‐variable case*

**Figure 16:**
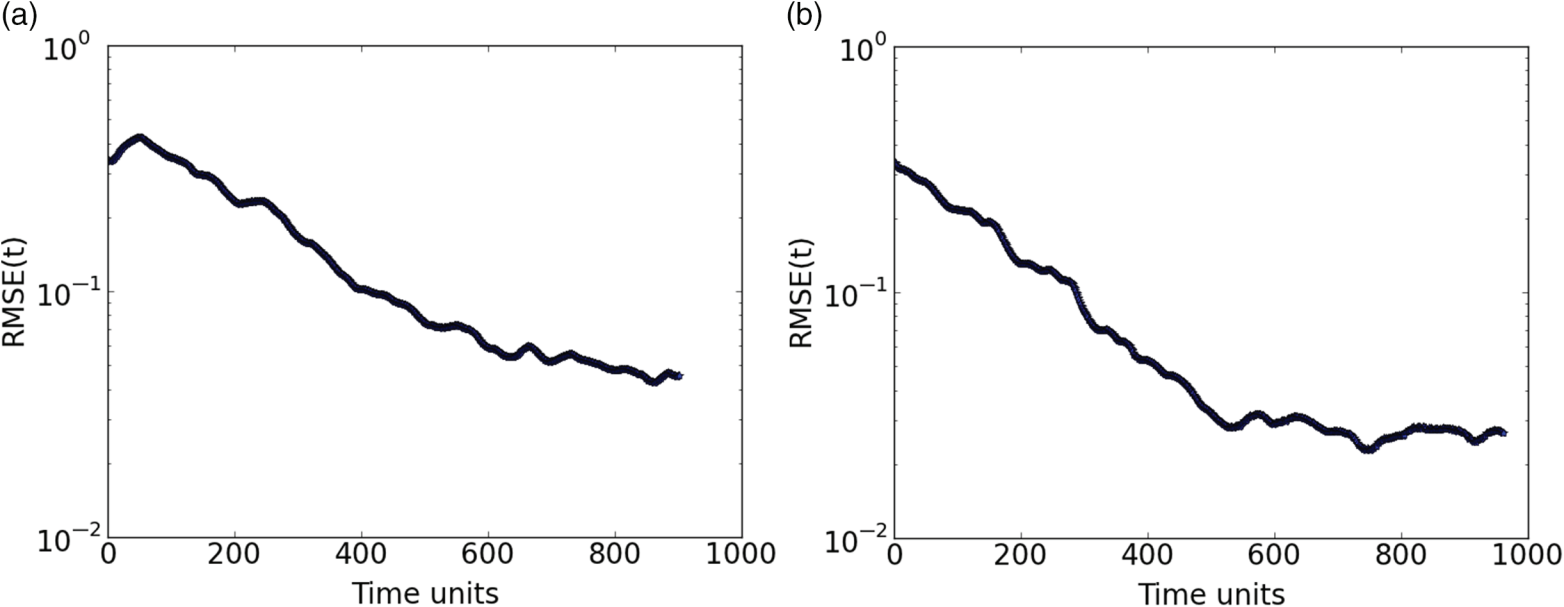
Synchronization error (RMSE) for a 1000‐variable system with 250 measured variables sampled equidistantly on the Lorenz'96 ring: (a) 100 ensemble members, without localization (D_d_=10), and (b) 20 ensemble members with localization applied (D_d_=5). Ensemble synchronization was used. [Colour figure can be viewed at http://wileyonlinelibrary.com].

For this experiment, 250 variables were observed for the same estimation period. As the number of variables increases, if we run the configuration without localization, the delay dimension needs to increase to *D*_d_=10 for synchronization to set in. In addition, the number of ensemble members required to get the system synchronized increases significantly to 100 members (Figure 16(a)).

**Figure 17:**
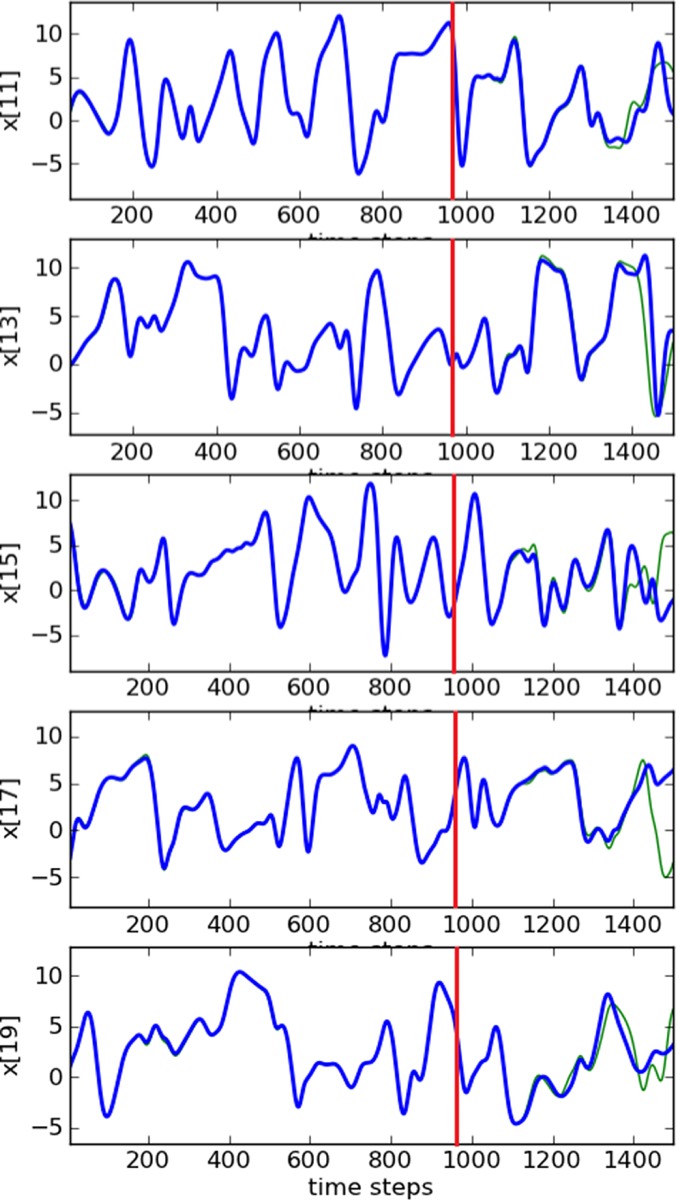
As Figure 15, but for five unobserved variables in a 1000‐dimension system for the ensemble‐based synchronization system with localization and 20 ensemble members. [Colour figure can be viewed at http://wileyonlinelibrary.com].

By using localization with a radius of influence *r* *a* *d*=10, the number of ensemble members is reduced from 100 to 20. Also, we are able to reduce the embedding interval, from *D*_d_=10 back to *D*_d_=5. Results (Figure 16(b)) are better than the ones without localization, producing good estimates and predictions (Figure 17).

At this point it is important to mention the computational complexity of the two methods, the pure synchronization (matrix propagation) and the ensemble‐based synchronization. The number of model runs needed is strongly reduced in the ensemble‐based method, the difference being a factor *D*_*x*_/*N*_ens_. Furthermore, the matrix method needs the linearized model equations, and these are typically more expensive than fully nonlinear model runs because more terms appear in the equations. It is worth mentioning that these time‐extended measurements aim to effectively extract information from other existing observations, at the expense of an additional computational complexity. If the system is fairly well observed, simpler methods can be used. However, if the system does not have enough available measurements, one must use a more computationally intensive algorithm, such as the one presented here.

Another interesting point to be added is the role of the variable *τ* in this system as an indicator of the temporal frequency of measurements needed for the achievement of accurate estimates and forecasts. To better understand this idea, an experimental simulation was performed (results not shown here), setting *τ*=Δ*t* and the window length equivalent to (*D*_d_−1)10Δ*t*. In this experimental configuration, the length of the window used is kept, but now all the measurements within it are used, instead of using observations at only every 10 time steps (the standard configuration used in the article). We found results which are roughly equivalent to the experiments shown here. That gives us an interesting clue about the minimum observation frequency needed to achieve successful estimates/forecasts. Therefore, our present configuration (e.g. using only the measurements that appear at every ten time steps inside the time embedding interval) seems to be enough. Using observations of these variables more frequently would not add much more information to this system.

## 4 Conclusions

An innovative methodology is proposed for an ensemble‐based synchronization scheme, which works well for a desirable small number of ensemble members. From a synchronization point of view, the initial values of the ensemble members were chosen in a random, isotropic distribution around the initial state. This helped the method to work, since the members are immediately attracted by the most expanding directions, so they represent, after a short transient, the unstable dynamics transversal to the synchronization manifold, which is what is needed for a stabilization method. This way, high‐dimensional applications of synchronization are now within reach. Different initial conditions and random‐number realizations were tested in all the cases presented here, deriving qualitatively and quantitatively similar results.

Table 1 shows the total RMSE means for the run periods, summarizing results for the 100‐ and 1000‐variable systems for the pure synchronization and the ensemble‐based synchronization with and without localization. It demonstrates that the synchronization scheme with a matrix propagation produces better synchronized systems with lower RMSEs than the ensemble‐based scheme. However, the former cannot be extended to higher‐dimensional systems, as a matrix the size of the system dimension has to be propagated by the linearized model. The ensemble method does provide good synchronization results, even for high‐dimensional systems when localization is used. Note that inflation is not needed in this type of framework.

**Table 1:** Total RMSE mean for 1000 time‐step runs for the pure synchronization (Synch) and the ensemble‐based synchronization (EnSynch).

		EnSynch	
*D*_*x*_	Synch	(no localiz.)	(with localiz.)
100	0.02	0.05	0.05^a^
1000	0.02	0.09^b^	0.05

a *N*_ens_decreased from 15 to 5.

b *D*_d_=10, in this case.

It is interesting to note the relation between the time embedding dimension and the number of observations at each observation time. As Rey *et al.* (2014a) noticed, to stabilize all growing modes off the synchronization manifold of a 20‐dimensional Lorenz'96 system, one could use an eight‐dimensional delay embedding of a single observable instead of (simultaneously) measuring eight different state variables of the system. This result was obtained for noiseless data. We found that, with noisy observations, increasing the embedding dimension *D*_e_=*D*_d_∗*D*_*y*_ by increasing *D*_d_ is not sufficient to achieve synchronization, but the number of observations *D*_*y*_ has to be chosen larger than one. We have not been able to synchronize the system when only one variable was observed, even when the observation error was small, of order 0.001, and *D*_d_ as large as 20, in the 20‐dimensional system. The only way to synchronize the system was to increase the number of observations at each observation time. In all simulations presented here *D*_*y*_=0.25*D*_*x*_ turned out to provide good results for *D*_d_=5. If we increase the number of available measurements to *D*_*y*_=0.5*D*_*x*_, we are able to synchronize the system with a delay dimension *D*_d_=2 (results not shown here), confirming this close relationship between *D*_d_ and *D*_*y*_. However, more research is needed to better characterize the observational requirements of the synchronization system.

Also, our findings indicate that the embedding dimension *D*_e_ needs to be of the order of the system dimension to obtain RMSE values below the observational error, but again, more research is needed. It should be noted that this result is related to the RMSE of the whole system, not just the observed variables. Another remark to be made is that in these experiments the results were not too sensitive to the value of the coupling constant *g*, as synchronization can still be obtained with *g*=1. We have used *g*=0.1 for slightly better results and so have reducing the influence of the whole coupling term in Eq. (5) by a factor of 10. Tuning this parameter may perhaps be trickier in a real geophysical system, and the search for an optimal value might need a variable dependent *g*. When the observation errors are large, we have to ensure that the synchronization will not lead to overfitting. This can be prevented by tuning the parameter *g*, such that the best forecasts are obtained. Finally, we did not investigate the influence of a non‐uniform observation network, combined with different localization radii, again an interesting endeavour for future work.

On the data assimilation side, our results suggest that both schemes are valuable tools to steer model states to the true evolution of the system. Nevertheless, although good estimates of the states are obtained, there are no uncertainties involved, so these methodologies should not be used as stand‐alone data assimilation methods. Furthermore, observations inside the time interval [*t*,*t*+(*D*_d_−1)*τ*] are used more than once, which would lead to complicated schemes in conventional data assimilation methods. Both problems, however, can be solved simultaneously by viewing them as part of a more comprehensive data assimilation method like a particle filter. This is indeed our final goal, to investigate the usefulness of these synchronization schemes as (part of) a proposal density in a fully nonlinear particle filter. van Leeuwen (2010) introduced a simple relaxation term to future observations in his particle filter to steer the particles towards the high‐probability region of the posterior between observations. This was incorporated in a high‐dimensional system in Ades and van Leeuwen (2015), and in a climate model by Browne and van Leeuwen (2015). The latter study found that the relaxation term was not functioning well enough, with the model drifting too far from the truth between observations. Browne (2016) found a similar issue, both with the simple relaxation term and with an Ensemble Kalman Smoother as proposal between observations. This is one of the motivations to investigate synchronization for this purpose in this article.

Since synchronization as discussed here is deterministic, the idea is to add a stochastic term to each synchronized particle, drawn from the model error pdf, so the scheme can be used in the particle filter framework. When the model error pdf is assumed to be Gaussian, as is most common because we do not know much about these errors, the proposal density related to synchronization is a Gaussian too, and easy to implement. Specifically, we need to change the weight of each particle by a factor

(18) $$\frac{p({\text{x}}_{i}^{n}|{\text{x}}_{i}^{n-1})}{q({\text{x}}_{i}^{n}|{\text{x}}_{i}^{n-1},{\text{y}}^{m})},$$

in which *i* is the particle index, *n* is the time index between observations and *m* is the observation time, in the case that observations are not available at every time step. $p({\text{x}}_{i}^{n}|{\text{x}}_{i}^{n-1})$ is the transition probability of the original model, and $q({\text{x}}_{i}^{n}|{\text{x}}_{i}^{n-1},{\text{y}}^{m})$ is the transition density of the modified model equation due to the synchronization term. Both of these transition densities are known and typically assumed to be Gaussian, e.g the first is a Gaussian with mean $f({\text{x}}_{i}^{n-1})$ and covariance **Q**. The implementation is similar to the relaxation‐term proposal in van Leeuwen (2010).

Finally, the use of time embeddings as a crucial factor in these schemes was discussed. Future work to be considered could be the implementation of a backward–forward (Pazó *et al.*, 2016) version of the synchronization code, in order to compare its efficiency and performance with the proposed methods.

## Appendix 1

### A Pseudocode for synchronization scheme

This appendix contains the pseudocode for the first six steps of the ensemble‐based synchronization scheme described in section 2.2.
